# Fully Printed Thermogalvanic Modules for Low-Grade Energy Harvesting

**DOI:** 10.1021/acsaem.5c02080

**Published:** 2025-08-26

**Authors:** Pedro Candiotto de Oliveira, Naveed ul Hassan Alvi, Najmeh Zahabi, Filippa Wentz, Kathrin Freitag, Lars Herlogsson, Ujwala Ail, Zia Ullah Khan, Igor Zozoulenko, Reverant Crispin, Dan Zhao

**Affiliations:** † Laboratory of Organic Electronics, Department of Science and Technology, 4566 Linköping University, SE-601 74 Norrköping, Sweden; ‡ Printed, Bio and Organic Electronics Units, Department of Smart Hardware, Digital Systems Division, 388792 RISE Research Institutes of Sweden AB, Södra Grytsgatan 4, 602 33 Norrköping, Sweden; § Wallenberg Wood Science Center, Department of Science and Technology, Linköping University, SE-601 74 Norrköping, Sweden

**Keywords:** low-grade thermal energy harvesting, screen-printing, thermogalvanic module, scalability, sustainability, thermogalvanic cell

## Abstract

Thermogalvanic cells offer a promising route for harvesting low-grade heat by utilizing temperature-dependent redox reactions at spatially separated electrodes. Their potential for low-cost, flexible, and sustainable energy conversion makes them attractive for scalable applications; however, practical implementation is limited by challenges in modular integration and manufacturability. Here, we report the development of a fully printed thermogalvanic module (TGM) that integrates screen-printed hybrid current collectors, activated carbon-based electrodes, an adhesive sealing layer, and a laser-drilled spacer. This fully additive and scalable fabrication strategy enables the precise assembly of complex architectures without traditional stacking or wiring. The resulting 36-cell TGM, employing widely available aqueous electrolytes, demonstrates a reproducible thermopower of 38 mV K^–1^ and a peak output power of 9 μW under a modest 14 K temperature difference. This work demonstrates a practical pathway toward large-area printed thermogalvanic systems for ambient heat harvesting and paves the way for future integration into flexible and wearable energy platforms.

## Graphical abstract

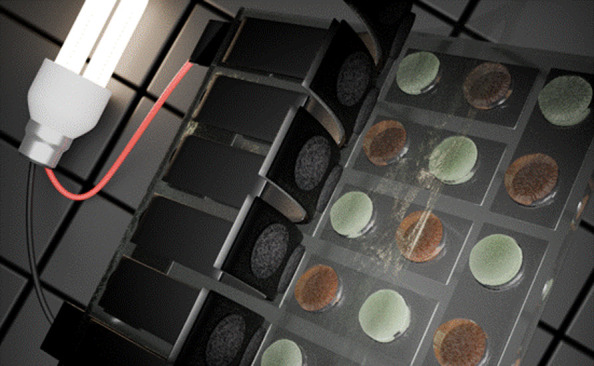

## 1 Introduction

In response to the escalating energy crisis and environmental degradation, the development of energy harvesters that generate clean electricity is becoming increasingly urgent. The abundant low-grade thermal energy on earth from solar heating, geothermal energy, and wasted heat has promoted research on heat-to-electricity harvesters.^1–7^ Thermoelectric generators can directly generate electric energy under a temperature gradient due to the strong coupling of heat flow and electric current materials. Various materials including metal alloys/oxides,^1^ conducting polymers,^7,8^ and hybrid conductors^9,10^ have been reported to possess thermoelectric properties in the past decades. However, the best-performing thermoelectric materials are based on BiTe alloys, which are expensive and challenging to process and dispose of.

Thermogalvanic cells (TGCs) exhibit heat-to-electricity converting property through reversible redox reactions on the electrodes at different temperatures.^1,6^ The best-performing electrolytes used in TGCs are aqueous, in which common redox couples exhibit high solubility and diffusion coefficients.^11–13^ Different approaches involving molecular technology were successfully applied to improve the Seebeck coefficient of TGCs electrolytes, such as redox entropy, phase transition, molecular recognition, and phase separation, as reviewed by Zhou et al. 2023. The increase in the Seebeck coefficient is due to an increase in the entropy difference in the redox species or creating a gradient concentration by the use of additives.^14^ In order to improve the sustainability of the whole cell, electrochemically active electrodes composed of abundant materials, such as conducting polymer^7^ and carbon-based composites,^15,16^ have been investigated as the electrodes in TGCs to substitute noble metals.

Although the typical redox electrolytes used in TGCs exhibit relatively high Seebeck coefficient (on the order of millivolt per Kelvin), the resulting output voltage is limited to only tens of millivolts with a moderate Δ*T* of 30–50 K. Similar to thermoelectric modules, thermogalvanic modules (TGMs) that connect multiple cells were developed to achieve practically useful output voltage and power. The manufacturing of TGMs involves fabrication of electrochemically active electrodes with complementary design, patterning of alternating p and n cells, and the assembly of different layers. Furthermore, effective sealing to avoid electrolyte drying is critical; otherwise, the power of the TGM will decrease due to increasing internal resistance and eventually disconnect. Multiple studies of TGM have been reported in the literature, mostly only as a final demonstration of the newly developed electrolytes with improved Seebeck coefficient.^2,11^ Moreover, due to the complex and nonscalable methods involved, the reported TGMs have so far been assembled by hand through manual connections of TGCs, making them unsuitable for large-area manufacturing.^2,17–21^

In this work, we developed a rational method for manufacturing TGMs utilizing the large-area screen-printing technology. Printable activated carbon (AC), commonly used in supercapacitors,^22,23^ is used as the electrochemical active electrodes in the printed TGM. A layer of commercial silver (Ag) ink together with a layer of conductive carbon composition (CC) were printed under the AC electrodes as the current collector to provide sufficient conductivity and avoid side reactions. Laser-drilled poly­(methyl methacrylate) (PMMA) was used as a spacer between the cells, which were sealed by the top and bottom electrodes using preprinted UV-cured adhesive. The fully printed 36-cell TGM was demonstrated with aqueous solution of potassium ferro/ferricyanide and iron­(II)/iron­(III) chloride and shows a reproducible thermopower of 38 mV K^–1^ and maximum output power of 9 μW (Δ*T* = 14 K). COMSOL simulations were used to further analyze the performance of the printed TGM and understand the properties of the TGM. The successful demonstration of a universal manufacturing prototype for low-cost and large-area production of TGMs complements the recent development of electrolytes with large Seebeck coefficient and paves the way for practical applications.

## 2 Experimental Work

### 2.1 Electrode Preparation and Electrical Characterization

Different current collectors were prepared by stencil printing (through an 80 μm thick precut plastic mask) of Ag ink CXT 0644 (Sun Chemical) and 7102 carbon conductive composition on the commercially available substrate from Skultuna Induflex, composed of a 10 μm thick copper layer insulated by a 60 μm thick PET layer. The printing was done on the plastic insulated layer. The printed substrate was dried in an oven at 110 °C for 15 min after each layer of printing. AC ink purchased from redox.me was stencil printed on the current collector for cyclic voltammetry (CV) characterization.

The conductivity of the current collectors was measured by s-302 Signatone four-point resistance probe equipped with a Keithley 2410 nanovoltmeter. For CV characterization, the printed electrodes on different current collectors were used as the working electrodes, a platinum gauze electrode was used as the counter electrode, and the reference electrode was Ag/AgCl/KCl (3.5 M). Two electrolytes were used: p-type 0.01 M potassium ferrocyanide trihydrate/0.01 M potassium ferricyanide in potassium chloride 1.0 M to suppress migration mass transport effects and n-type 0.01 M iron­(II) chloride tetrahydrate and 0.01 M iron­(III) chloride hexahydrate, with 1.0 M potassium chloride added as well. The CVs were performed at the scan rates of 1, 5, 10, 15, 20, and 50 mV s^–1^ for both electrolytes. For the p-type potassium ferro/ferricyanide, the electrochemical window was from 0 to 0.60 V, while for the n-type iron­(II)/iron­(III) chloride, it was from 0 to 0.75 V due to the different formal potentials for each of the redox pairs.

### 2.2 Fabrication and Characterization of TGCs

An Ag/CC current collector was first printed through an 80 μm thick plastic stencil mask (10 mm wide and 30 mm long) on Skultuna Induflex. An AC electrode was then printed on the middle of the current collector (10 mm diameter). A cavity of 5 mm diameter was drilled in 2 mm thick polydimethylsiloxane (PDMS), which was glued onto the substrates with electrodes to assemble single-cell TGCs (depicted in Figure S1a).

For the assembly of the 2-cell TGC, Ag/CC of 10 mm wide and 45 mm long was printed as the bottom layer current collector and connection between the two cells, and 30 mm wide and 10 mm long as the top current collector. The AC electrode was then printed (10 mm diameter) in the center of the double current collector layers. Two cavities of 5 mm diameter each were drilled in 2 mm thick PDMS, which was glued onto the substrates with electrodes to assemble the 2 cells TGM, i.e., one thermocouple. Figure S1b shows all of the layers and structure in detail in a cross-section illustration of the device. The electrolytes utilized for the TCGs and all TGMs were p-type: 0.2 M potassium ferrocyanide trihydrate/0.2 M potassium ferricyanide, and n-type: 0.05 M iron­(II) chloride tetrahydrate and 0.05 M iron­(III) chloride hexahydrate.

For thermoelectric characterization, multiple temperature differences were applied between the two electrodes by Peltier elements for the three different cells. The voltage between the electrodes was monitored by a Keithley 2182A nanovoltmeter, and two thermocouples monitored the temperature at each electrode. The electrochemical impedance spectroscopy (EIS) measurements were performed at open-circuit potential, with 5 mV amplitude, and in the frequency range between 200 kHz and 100 mHz.

### 2.3 Screen-Printing of TGMs

A screen-printer DEK Horizon 03iX that provides an alignment accuracy of ±12.5 μm was used for printing all of the layers in the TGM. Figure S2a shows a picture of the DEK Horizon 03iX screen-printer utilized, and Figure S2b shows a schematic representation of the screen-printing process using a DEK screen printer. All printing steps were performed under ambient conditions. Three different standard polyester screen meshes were utilized. The screen mesh count was 120 threads per cm for the current collector layers, 77 for the electrode layer, and 36 for the glue layer. The screen thread diameter was 34 μm for the current collectors layers, 48 for the electrode layer, and 100 for the glue layer. The print gap varied between 2 and 4 mm, and the squeegee pressure varied between 12 and 20 kg for the printed layers.

In the first step, a Ag/CC composite layer was printed onto the PET side of the insulated copper foil substrate. This conductive layer consisted of a carbon layer deposited on top of a printed silver layer, where silver ink (CXT 0644, Sun Chemical) was printed for the silver layer. Carbon ink (7102, DuPont) was printed on top of the silver layer. Two different masks (Figure S3a,b) were employed to define the bottom and top current collectors, which, upon assembly, form 36 cells connected electrically in series and thermally in parallel. The printed conductive layers, with a final thickness of 16 μm, were thermally cured in a convection oven (Natgraph) at 120 °C for 2 min.

The electrode layer composed of the AC from redox.me was then printed over the predefined current collectors. The electrode layer was dried at 110 °C in a convection oven (Natgraph) for 2 min. Finally, UV-curable adhesive (Kiwo UV 94, KiwoPrint) was printed over each structure to enable attachment of the patterned substrate to 2 mm thick laser-cut PMMA, which was patterned to match the electrode structure. The adhesive was UV-cured in a conveyor belt system, ensuring a leak-proof seal while maintaining good contact between the electrode/electrolyte interfaces.

### 2.4 TGM Assembly and Characterization

The 36-cell TGMs were assembled after the screen printing of all of the layers. The PMMA spacer was positioned and pressed over the bottom electrodes. The precise amount of 37 μL of electrolyte was filled into the chambers, alternating between p and n types. Finally, the top layer was positioned and pressed over the filled PMMA chambers, sealing the TGM. The assembly of the modules was done with the help of an alignment tool developed and built in-house, which together with printed alignment marks in the Skultuna substrates and laser-drilled marks in the PMMA separator allowed reproducibility, better contact between the electrodes and electrolytes, and proper alignment between the substrates and the PMMA. The stability test of TGM was done by applying a constant and continuous temperature difference of 14 K (23 °C on the cold side and 37 °C on the hot side) for 10 days, while measuring the output voltage. For flexible TGM, styrene ethylene butylene styrene (SEBS) film of 1 mm thickness was used as a spacer instead of PMMA.

### 2.5 COMSOL Simulation

Three-dimensional finite element analysis was conducted using COMSOL Multiphysics 6.3 to simulate the thermoelectric behavior of the system. The analysis followed a structured approach, beginning with the creation of a 3D geometric model within software. Subsequently, a meshing procedure was applied, utilizing sweep mesh and scale mesh techniques to ensure the computational efficiency and accuracy. Following meshing, the material properties and boundary conditions were assigned. The material properties utilized for the simulations are presented in Table S1. Finally, the solver computed the thermoelectric profile of the TGM system.

The simulation was performed under steady-state conditions. To simplify the computational model while maintaining fidelity to real-world scenarios, several assumptions were adopted. All surfaces, except for the heat source and heat sink, were considered adiabatic, implying no heat exchange with the surroundings. Electrical and thermal contact resistances were negligible. Heat losses due to convection and radiation from all surfaces were disregarded. The heat source and heat sink were modeled as thermal boundary conditions with fixed temperature values. All material properties, including electrical conductivity, thermal conductivity, density, and the Seebeck coefficient, were assumed to be spatially uniform. The TGM was cooled by a heat sink attached to a cold surface.

The physical mechanisms governing the thermoelectric system involve the coupling of thermal and electrical fields, which are characterized by the thermoelectric effect. Heat transfer within the solid components and fluid domains were modeled using the heat transfer module. The heat conduction equation governing the thermoelectric analysis is given by eq 1.^24^

1 $$\rho c_{p}\nabla T+\nabla \cdot \left(-k\nabla T\right)=Q$$

where ρ is the material density, *c*_p_ is the specific heat capacity at constant stress, *T* is the temperature, *k* is the thermal conductivity, and *Q* represents Joule heating.

To model the electrical behavior of the TGM system, the electric current module was applied to conductive components. The governing equation for electric current density is described in eq 2.^25^

2 J=σE−σS∇T

where *J* is the electric current density, σ is the electrical conductivity, *E* is the electric field intensity, *S* is the Seebeck coefficient, and ∇T represents the temperature gradient.

## 3 Results and Discussion

### 3.1 The Selection of Printable Current Collectors

A typical TGM is built by connecting multiple cells electrically in series and thermally in parallel. In this configuration, the total resistance of the module depends on the resistance of each cell, as well as the electrical resistance of the connection between the neighboring cells. Electrodes in a TGC should have relatively high conductivity and allow efficient electron-transfer to and from the redox molecules in the electrolytes. The AC can be screen-printed to form a porous electrode in TGC that provides sufficient electrochemical activity.^26^ However, the electrical conductivity of the AC electrode is only 1.4 S cm^–1^, which could introduce additional resistance into the module. Hence, it is important to preprint current collectors before the AC electrode that facilitate the reversible redox reactions with the redox molecules.

To select a suitable current collector, electrodes with different current collectors were characterized in potassium ferro/ferricyanide solutions. First, commercial Ag ink was printed underneath the AC ink to form a highly conductive layer as a current collector. As summarized in Table 1, the conductivity of the printed Ag layer is 6 × 10^5^ S cm^–1^, which is over 5 orders of magnitude higher than that of the AC layer (1.4 S cm^–1^). However, irreversible side reactions (indicated by the peaks appearing close to 0 V) were observed in the CV plot shown in Figure 1a. This could be ascribed to the porosity of the AC that allows redox electrolyte to penetrate and react with the silver layer.^16,27^

**Table 1:**
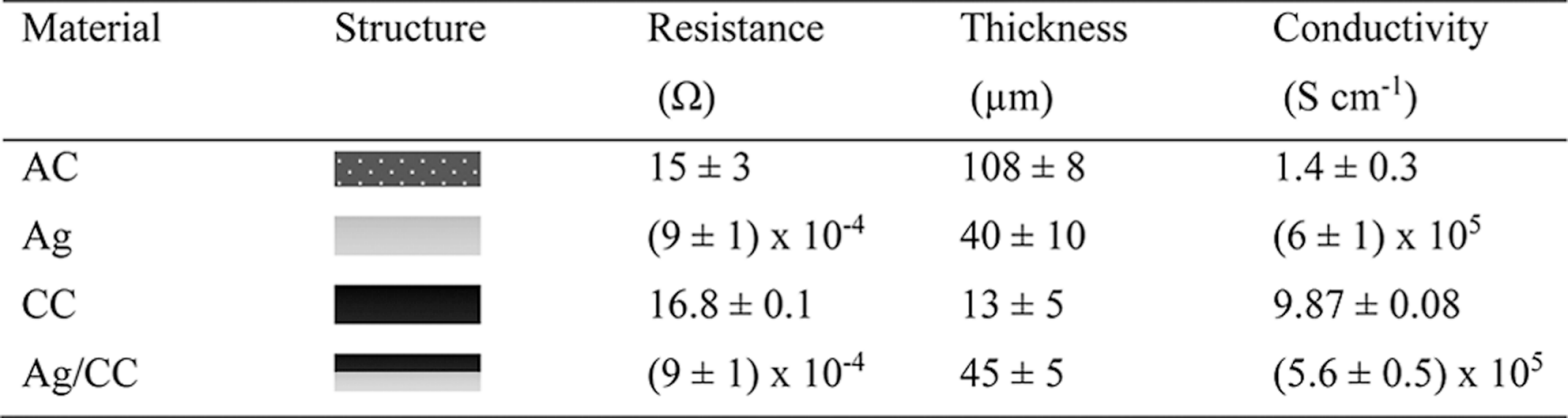
Properties of Current Collector and Electrode Materials Used in Figure 1

**Figure 1:**
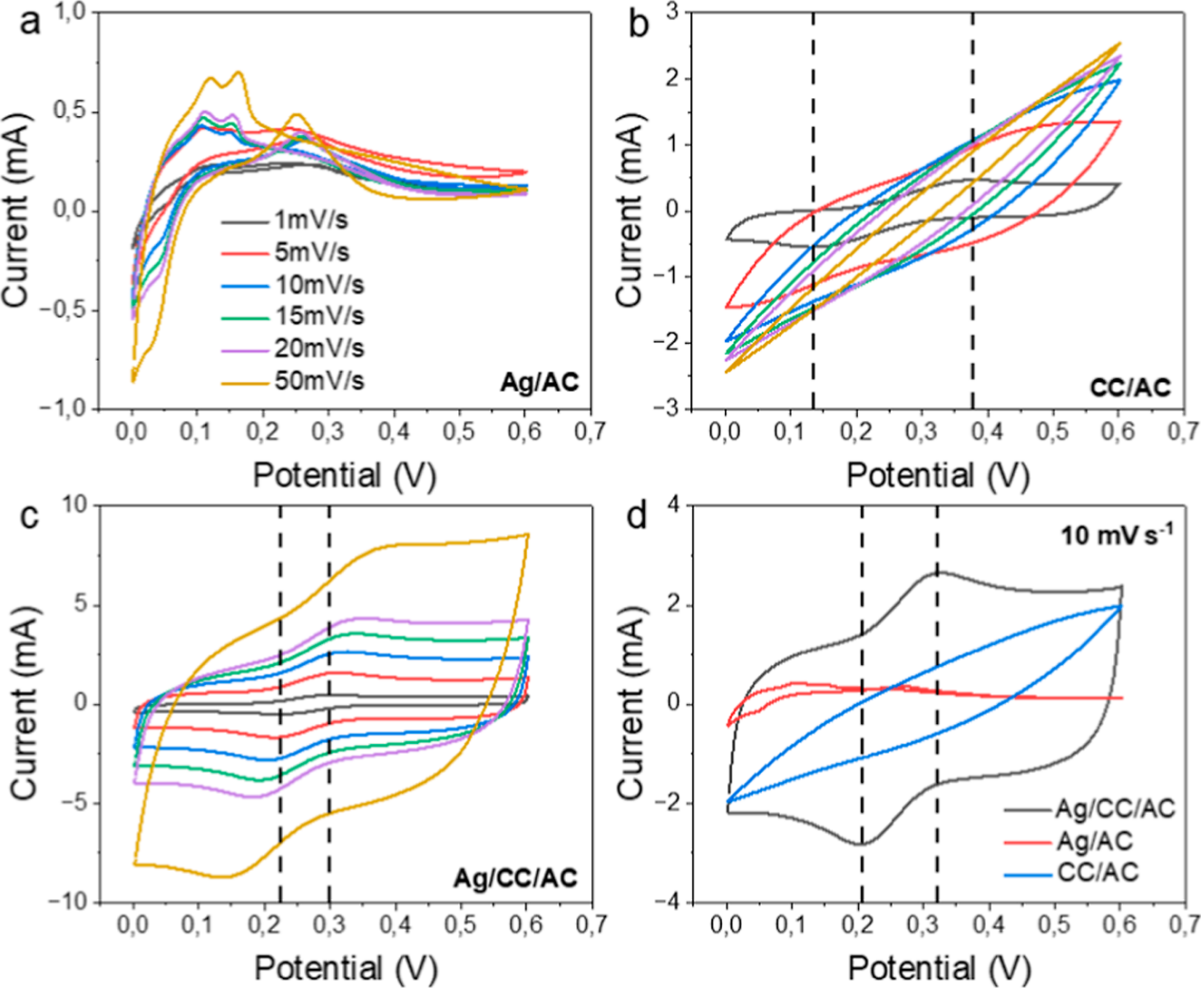
Electrochemical characterization (CVs) of AC-printed electrodes in the ferro/ferricyanide electrolyte in different current collectors. (a) Ag. (b) CC. (c) Ag/CC. The same legend in (a) applies to (b,c). (d) Comparison of the Ag/CC/AC, Ag/AC, and CC/AC systems at a 10 mV s^–1^ scan rate. The vertical dash lines indicate the redox peaks of the active electrolyte.

Another inert nonporous conductive ink, DuPont 7102 CC, was printed as the current collector under the AC electrode. As shown in Figure 1b, the expected peak current related to the electron transfer between the redox ions and the electrode could be observed at the lowest scan rates (indicated by the dashed lines in Figure 1b). However, the inclination of the voltammograms increases with the scan rate due to the highly resistive behavior of the CC. Indeed, the conductivity of the electrode with CC as the current collector is only slightly higher than the AC (9.87 S cm^–1^).

Considering the characteristics of different materials, we designed a hybrid current collector with both Ag and CC. The Ag layer was printed first to provide high electrical conductivity, which was covered by a second layer of inert CC to avoid corrosion. The conductivity of the Ag/CC current collector was largely improved to 5.6 × 10^5^ S cm^–1^ compared to only CC. Moreover, the oxidation and reduction characteristic peaks from the potassium ferro/ferricyanide redox reaction^7,13^ could be identified for all scan rates (Figure 1c). The box-like shaped CV curves with low inclination also confirmed the relatively low resistance of the electrode with the hybrid current collector.^28,29^

The comparison of the voltammograms for three different current collectors at the same scan rate of 10 mV s^–1^ in Figure 1d clearly shows that the Ag/CC current collector greatly improved the electrochemical performance of the electrode. The same characterization was performed in aqueous solution of iron­(II)/iron­(III) chloride, which is typically used as the n-type electrolyte in TGCs. The CV curves shown in Figure S4 indicate the same conclusion: the combination of Ag/CC is the most suitable current collector. The application of Ag/CC current collectors is important to ensure sufficient conductivity and stability for the electrode in TGMs.

### 3.2 The Performance of Individual Printed TGCs

After selecting the suitable current collector of the electrodes, the performance of printed p- and n-type TGCs was investigated. Electrodes composed of a Ag/CC current collector and AC electrodes were manually printed on the plastic side of the insulated Skultuna copper foil. The TGC was assembled by sandwiching a precut PDMS chamber (5 mm diameter and 2 mm thickness) with two identical electrode/collector assemblies. Electrolytes containing potassium ferro/ferricyanide or iron­(II)/iron­(III) chloride were injected into the chamber.

The working principle of TGCs is illustrated in Figure S5. The Seebeck coefficients of the cells were obtained by linearly fitting the measured thermal voltage vs the applied Δ*T* (Figure S6). As summarized in Figure 2a, the Seebeck coefficient is −1.15 ± 0.01 mV K^–1^ for potassium ferro/ferricyanide and +1.08 ± 0.03 mV K^–1^ for iron­(II)/iron­(III) chloride, similar to previous reports.^30–34^ The Seebeck coefficient of one thermocouple composed of one p- and one n-cell (Se­(TC) = |Se­(p)| + |Se­(n)|) is 2.20 ± 0.02 mV K^–1^, as the expected sum of the two individual cells (2.23 mV K^–1^).

**Figure 2:**
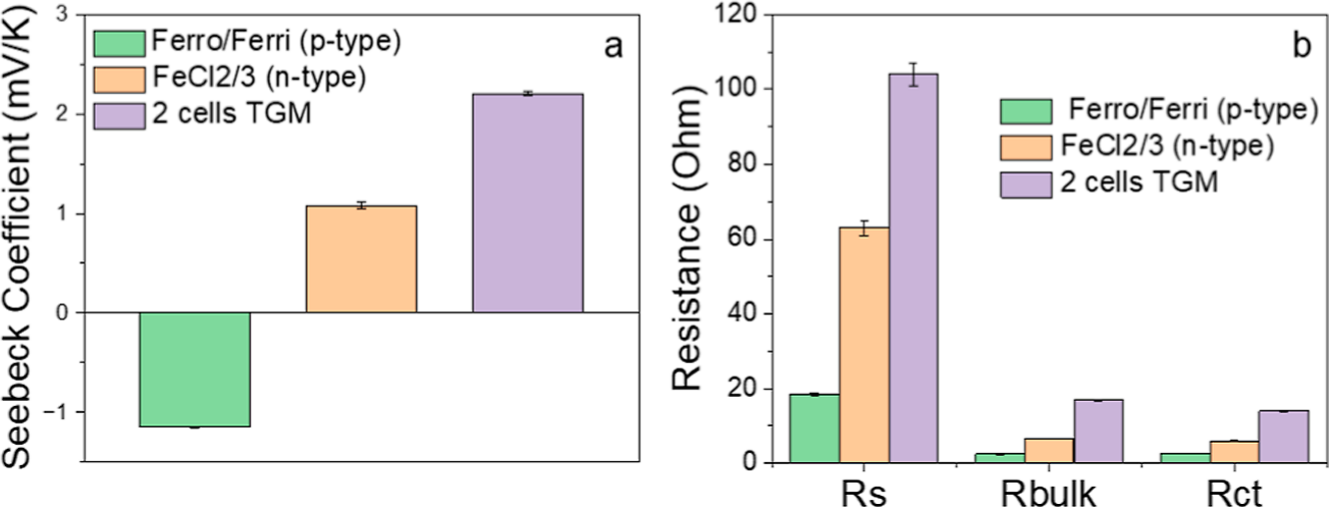
Characterization of p and n TGCs and 2 cells TGM. (a) Seebeck coefficient. (b) EIS resistances decomposed. The error bar is the result of three measurements on the same device.

The contribution of different resistances of the n and p-type TGCs was characterized by EIS. Figure S7 shows the Nyquist plots for the individual p- and n-cells and the thermocouple. After fitting each EIS spectrum with the equivalent circuit, shown in the inset of Figure S7a, utilizing the software RelaxIS, the values of series resistance (*R*_s_), charge transfer resistance (*R*_ct_), and bulk resistance (*R*_bulk_) are presented in Figure 2b. The value of *R*_s_ that includes the electronic resistance of the electrode and ionic resistance of electrolyte is lower for ferro/ferricyanide cells (18.5 Ω) compared to the iron­(II)/iron­(III) ones (63.0 Ω). This is mainly due to the different ionic conductivities of the electrolytes. The values of *R*_ct_ and *R*_bulk_ are considerably low for both electrolytes (below 10 Ω). This indicates that the process of charge transfer and the mass transport of the redox ions is fast in TGCs with printed electrodes. The obtained *R*_s_ value for a pair of cells is 104 Ω, slightly larger than the sum of the individual TGCs, which could be due to the connection between the p- and n-type cells. The values of *R*_ct_ and *R*_bulk_ for the p–n thermocouple are larger compared to the sum of the individual cells while still below 20 Ω. The EIS results of p, n cells and the thermocouple showed that the n-type cell (iron­(II)/iron­(III) chloride) limits the resistance of the TGM. It is important to develop n type of electrolyte of improved ionic conductivity, charge transfer, and diffusion properties. Additionally, it is possible to adjust the dimension ratio of p and n cells in a TGM to balance their resistance for further optimization.

### 3.3 The Printing of TGMs

The printable module was designed with 36 cells, considering the resolution in practical printing and the scale of generated energy. The layout of the complete module is illustrated in Figure 3a. First, current collectors composed of Ag/CC double layers were printed using complementary screen masks for the top and bottom electrodes. Figure 3b shows the bottom silver layer printed on top of the Skultuna substrate. The mask was designed with alignment marks and margins for standardization of printing and assembly of the TGMs. As shown in Figure S8a, 6 devices could be printed on each substrate with the commercial screen. Two layers of AC ink were printed on top of the current collector to form the active electrode using a second mask. The electrodes are 5 mm diameter circles that are equally spaced, each pair centralized in the rectangle-shaped current collectors. A third mask was used to print the adhesive layer that glued the substrate with electrodes to the separator. The design of the adhesive mask was the negative of the electrode mask, ensuring effective sealing of the chamber to avoid electrolyte leaking. Figure 3c shows the photo of the bottom side of the TGM with the printed hybrid current collectors, electrodes, and glue.

**Figure 3:**
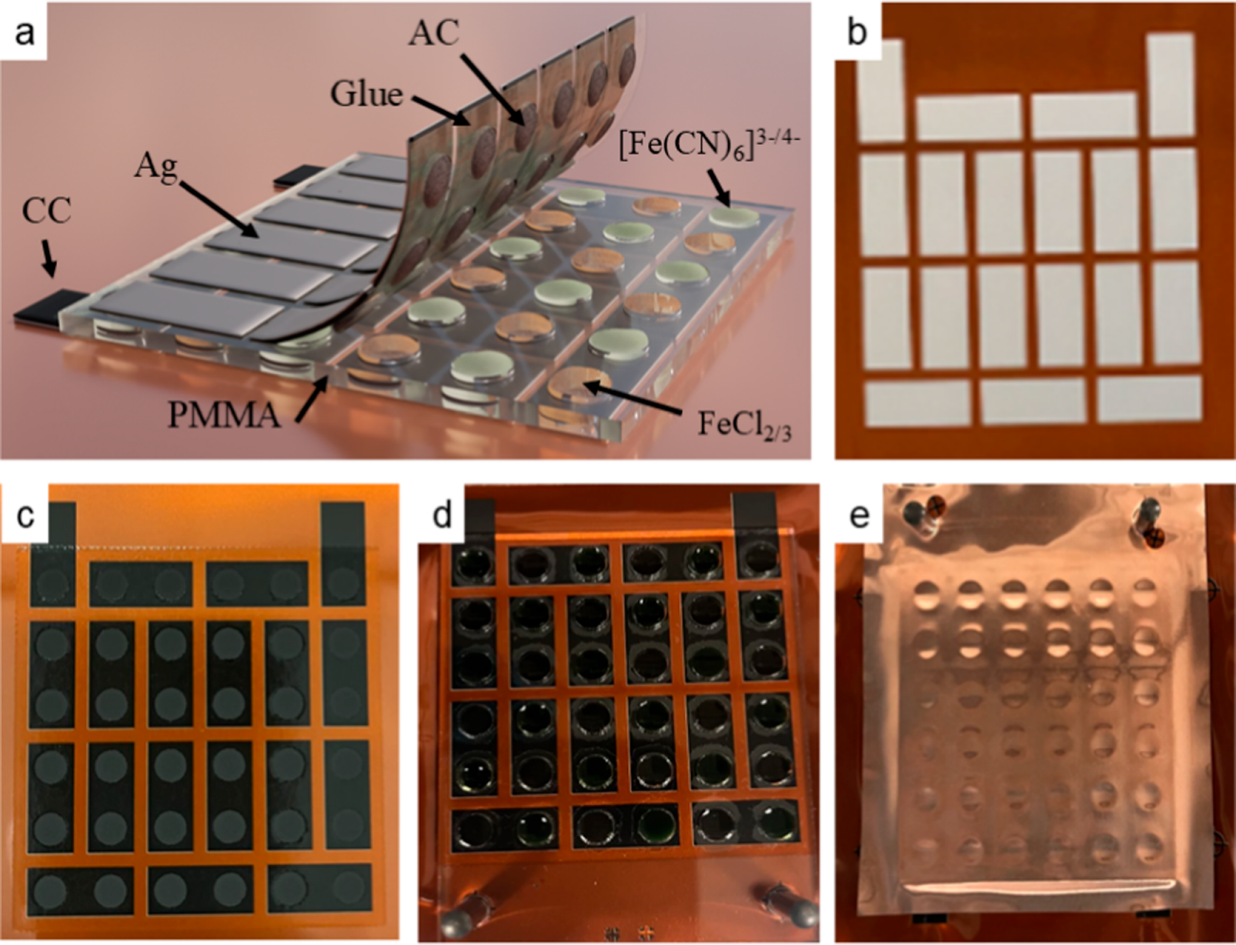
TGM (a) illustration scheme of the TGM with all the components: Ag/CC current collector, AC electrodes, UV cured glue, PMMA chamber, and electrolytes of potassium ferro/ferricyanide and iron­(II)/iron­(III) chloride. Photo of the (b) Ag layer of the current collector, (c) AC electrodes and glue on top of the hybrid current collector, (d) PMMA on top of the printed layers, and (e) a complete assembled TGM.

PMMA was selected as the spacer here because of its low-cost, easy accessibility, and high water barrier. Importantly, PMMA can be processed with the laser cutting technology to drill the designed cavities for cells in TGMs. As shown in Figure S8b, 36 cavities with 5 mm diameter were laser drilled using the same design as the AC electrode. Two additional holes were drilled on the bottom of the PMMA spacer to facilitate alignment when assembling the top electrode on the filled chambers. The drilled PMMA frame was glued on the bottom electrodes on the margins of each AC electrode (photo shown in Figure 3d). Here, the p and n cells are filled with an aqueous solution of K_3/4_[Fe­(CN)_6_] and FeCl_2/3_ in standardized amounts (37 μL per cell). The accurate amount is critical to enable sufficient contact between electrodes and electrolytes on both sides of the module and avoid leakages during the assembly of the devices. As the final step, the top electrodes were sealed on the PMMA with the help of a custom-built alignment jig. Figure 3e shows a picture of an assembled TGM. Two mm thick PMMA separator (corresponding to the thickness of the module) was used here in order to achieve relatively large Δ*T* while not being too bulky. Figure S9 shows the cross section of the TGM with the thickness of each layer.

### 3.4 The Performance of Printed TGMs

The Seebeck coefficient of the 36 cell TGM was determined in the same way as for the single cell TGCs and the 2-cell thermocouple. As shown in Figure 4a, the linear fitting of the output voltage versus Δ*T* for 4 devices of the same architecture gives a reproducible thermopower of 38 ± 3 mV K^–1^. The theoretical thermopower obtained from simply multiplying the Seebeck coefficient of 18 thermocouples (2.20 mV K^–1^) is 39.6 mV K^–1^. COMSOL simulations shown in Figure S10a,c confirmed that the thermopower of the TGM was expected to be 40.7 mV K^–1^. The printed TGM could generate 96% of the theoretical thermopower, which proves the success of the manufactured prototype.

**Figure 4:**
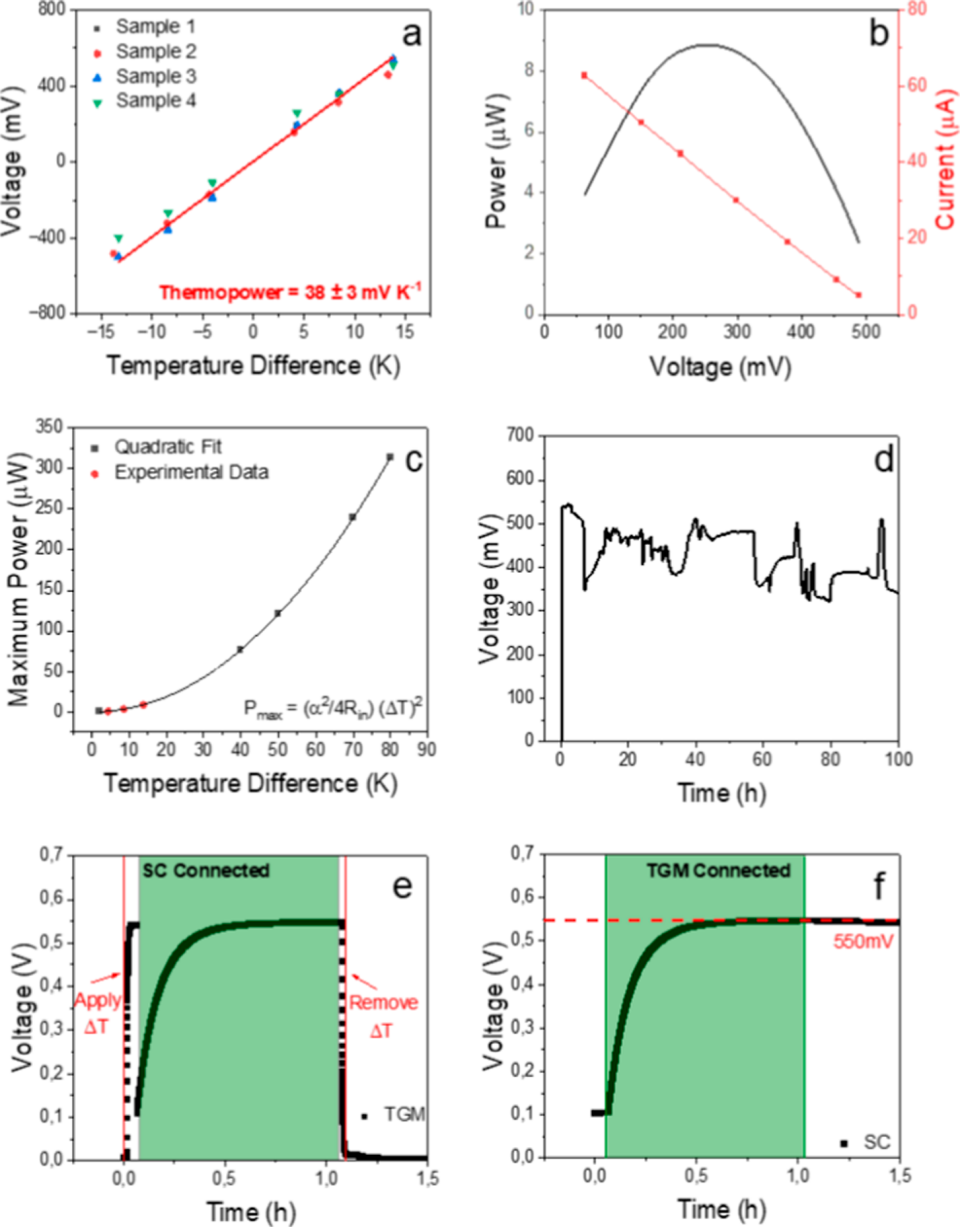
Characterization of TGM with 36 cells: (a) thermopower. (b) Power and current outputs (maximum power = 9 ± 1 μW). (c) Maximum power output – measured and simulated. (d) Stability test ran for 100 h. (e) TGM voltage while charging supercapacitor. (f) Supercapacitor voltage while being charged by the TGM. The red line in (a) indicates the linear fitting of the data.

The evolution of the resistances with the increased number of cells in the module was investigated by EIS. As shown in Figure S11 and Table S2, the internal resistance of the printed TGM is 2204 Ω, which includes series resistance (1010 Ω), charge transfer resistance (264 Ω), and bulk resistance (930 Ω). The increase in *R*_bulk_ indicates that scaling up the number of cells in TGMs could affect the mass transport, which is mainly responsible for the increase in the total resistance. Hence, it is important to find a balance between the number of cells and total resistance in order to obtain sufficient output voltage power. The output current and power of one TGM with Δ*T* of 14 K is presented in Figure 4b, with a maximum power of 9 ± 1 μW.

The maximum current shown in Figure 4b was 63 μA, and the current at maximum power is 42 μA, which approaches suitable values to integrate and power systems of low demanding current, for instance remote systems for monitoring, or sensors.^35^ The sufficient conductivity of the current collector is important for reaching a high output power. As comparison, the TGM using only CC as the current collector shows a much lower normalized maximum power (5 nW m^–2^ K^–2^) compared to the one with Ag/CC current collector (64 nW m^–2^ K^–2^) (Figure S12). As shown in Figure 4c, the measured maximum output power of the printed TGM (red dots) increases quadratically with Δ*T*, following *P*_max_ = (α^2^/4*R*_in_)­(Δ*T*)^2^ (in which α and *R*_in_ are the thermopower and internal resistance of the TGM). Hence, the output power of the TGM could be enhanced by applying a larger Δ*T*. As indicated by the calculated data (black squares) in Figure 4c, the maximum power output of the same TGM could increase to over 300 μW when Δ*T* is 80 K, showing great application potential of the printed device.^2,36^

The long-term stability of the printed TGM was tested by continuously applying an Δ*T* of 14 K. As shown in Figure 4d, the output voltage started at around 550 mV when Δ*T* is applied, as expected according to the Seebeck coefficient. Small variation (∼10% of the expected thermal voltage) in the output voltage took place after 10 h, possibly due to water evaporation with time or small oscillations from the Peltier elements that provide Δ*T*. It is important to notice that output thermal voltage remained above 75% after 100 h of operation under heating (the temperature of the hot side of the TGM is 31 °C). Even after 240 h of continuous measurement, the TGM could still generate an output voltage of around 150 mV (Figure S13). The long-term operation for aqueous TGM is ascribed to the effective sealing with metal-coated substrates and waterproof UV glue. The stability performance of the printed TGM is in the same order as the best in the literature (77% retention after 7 days reported by Kim et al.).^37^

The performance of the printed TGM is compared with those of the other reported TGMs in the literature in Table S3. The high output voltage and power of some reported TGMs are mainly due to the improved electrolytes, while the cells are individually prepared and manually connected into a module. For instance, the work of Han and Xie in 2023 ^2^ reported an individual cell Seebeck coefficient of 10 mV K^–1^ and the one of Duan et al. in 2018 ^11^ reported a Seebeck of 4.2 mV K^–1^. Interestingly, Duan et al. in 2019 reported a modified *I* ^–^/*I*_3_^–^ electrolyte that can have its Seebeck tuned from +0.71 mV K^–1^ to −1.91 mV K^–1^, by adding a nanogel to it.^36^ The TGM reported in this work utilizes simple aqueous electrolytes with limited Seebeck coefficient while still presenting a comparable performance. The use of electrolytes with higher individual Seebeck coefficients can increase the output voltage of the TGM, while keeping the same number of legs. Moreover, the use of gelled or solid-state electrolytes would lead to fully leakage-free devices, increasing their stability and potential effective area.

In order to extend the potential for practical applications, we explored the possibility of using printed TGM to charge a commercial supercapacitor (SC). The SC S-Power 2R (produced by Ligna Energy AB) with capacitance of 50 mF was directly connected to the TGM exposed to Δ*T*. As shown in Figure 4e, the thermal voltage generated by TGM stabilized at 550 mV when submitted to an Δ*T* of 14 K. The output voltage of the TGM experienced a sudden decrease when connected to the SC, which is equivalent to connecting a small load resistance. The output voltage slowly increased until it reached the initial value of 550 mV. Meanwhile, the measured potential of the SC (Figure 4f) increases in the same trend from its discharge state of 100 mV (due to the residual charge).

In the charging process of SC, the output voltage of the TGM increases, and output current decreases, following the output curve in Figure 4b. The beginning of the charging is equivalent to connecting a small load resistance to TGM, which is characterized by small output voltage and large current. As the potential of the SC increases, the equivalent resistance of the SC increases, which leads to a decrease in the charging current but increasing output voltage of the TGM. The current continuously decreases when the potential of the SC approaches the thermal voltage of the TGM. After around 30 min, the SC was charged to the thermal voltage generated by the TGM of 550 mV. The averaged charging current of 15.3 μA can be calculated by $I_{av}=\frac{CV}{t}$ . The successful charging was confirmed by continuously tracking the voltage of the SC after disconnecting from the TGM. As indicated by the dashed line in Figure 4f, the potential of the SC maintained at around 550 mV even after 1 h when the TGM is disconnected.

A SC with a large capacitance of 1.2 F (S-Power 2S from Ligna Energy) could also be charged by printed TGM. As shown in Figure S14, the potential of the SC almost reached the thermal voltage of the TGM exposed to Δ*T* of 14 K after 9 h of charging. The extended charging time is due to the 20 times larger capacitance compared to the SC shown in Figure 4f. The averaged charging current for this SC is 20.4 μA, considering a charging time of 9 h. Relatively good potential retention of the charged SC was observed by monitoring the potential for 24 h after disconnecting from the TGM (Figure S14b). The possibility of charging a commercial supercapacitor highlights the promising application of using the printed TGM for collecting energy from low-grade heat.

### 3.5 Flexible TGM Prototype

After successfully demonstrating the printing of complete TGMs, we extended the manufacturing prototype to flexible devices. Flexibility is important for TGMs to form a tight contact with the heat source to maximize the harvesting efficiency. Here, we used SEBS as the separator and container for electrolytes in TGMs. SEBS is a hydrophobic polymer which easily forms flexible self-standing films that can be laser patterned as PMMA and glued with the UV-cured glue to the printed layers.^38^ The Seebeck coefficient of the flexible TGM in a planar configuration is 35 ± 5 mV K^–1^ (Figure 5a), which is very similar to the one of the PMMA-based 36 cells device.

**Figure 5:**
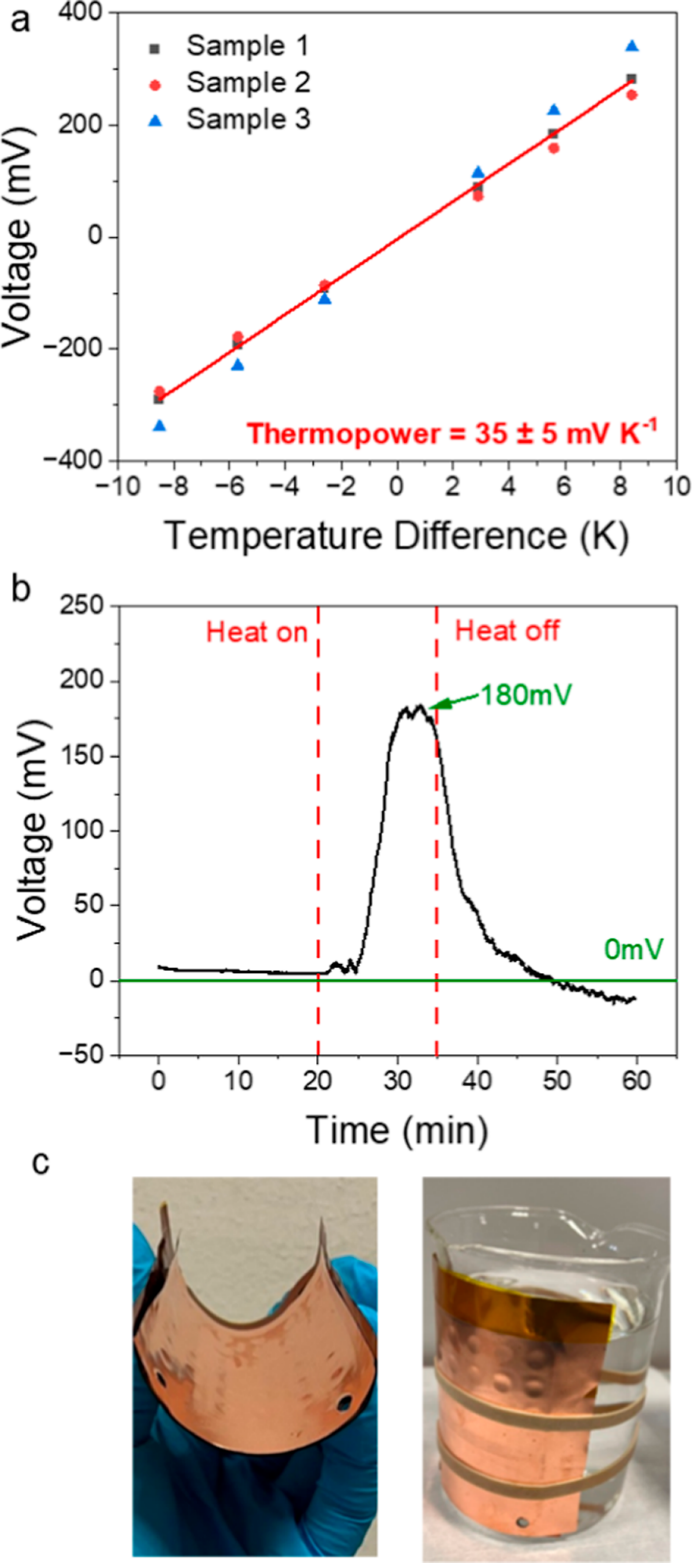
Characterization of bendable TGM with 36 cells. The red line in (a) indicates the linear fitting of the data. (a) Thermopower of the module in the flat configuration. (b) Output voltage of the module in the bent configuration, as proof of concept. (c) Pictures of the bent TGM in hand and wrapped around a beaker.

The function of the flexible TGM was demonstrated by harvesting energy from hot water in a beaker. The hot plate was turned on after recording the voltage of the bent module for 20 min, and the water inside of the beaker was heated to 50 °C. The measured output voltage gradually increased from 0 mV to 180 mV due to the established Δ*T* between the two sides of the TGM (Figure 5b). The heating was kept on for 15 min, and a thermovoltage of 180 mV was recorded. After removing the heat, the measured voltage recovered to its original value, as in the initial rest state. Figure 5c shows that the TGM with the SEBS spacer shows good flexibility, both bent in the hand and attached to a beaker with a radius of 70 mm, where the demonstration exhibited in Figure 5b was obtained.

This shows the possible application of printed TGMs on a curved surface. This is an important step toward the integration of TGMs into wearable devices for converting heat from the human body.^35,39,40^ The flexibility of the printed TGM is currently limited by the substrates and electrodes that are not stretchable; further exploration of those components could improve the flexibility of the TGM.

## 4 Conclusions

In this article, we developed a rational manufacturing method for low-cost and scalable production of TGMs based on the combination of high-throughput printing technologies, laser-assisted drilling, and a simple assembly through the printed adhesive patterns. Unlike conventional thermoelectric module designs, our approach utilizes an electrically insulated metal foil as a thermally conductive substrate in place of ceramic plates, offering both cost-effectiveness and flexibility. On top of that substrate, we printed interconnects consisting of a dual layer made of a bottom layer of Ag ink (for its high electrical conductivity) and a top layer of CC carbon ink (for its compact and chemically inert nature). Finally on those interconnects/collectors, a round AC electrode was printed for each cell, providing a large-surface area with the electrolytes and promoting low electron transfer resistance between the redox electrolyte and the electrode.

The performances of printed single p- and n-cells or p–n thermocouples and TGM with 36 cells were investigated and compared to COMSOL simulations. Scaling up the number of cells in TGM effectively multiplied the Seebeck coefficient up to 38 mV K^–1^, which corresponds to 96% of the calculated value. The series and contact resistances in the printed TGM increased with the number of cells, as expected. On the other hand, the bulk resistance had a substantial increase, being the limiting resistance of the device and impacting mostly on the output current and power. The maximum output power of the printed TGM reaches 9 μW with applied Δ*T* of 14 K, and the TGM maintained 75% of the output voltage after continuous operation for 100 h.

Moreover, we demonstrated the possibility of producing a flexible TGM using the same manufacturing prototype. The manufactured prototype of printed TGM reported in this work perfectly complements the materials development in the field of TGCs. The combination of the printed TGM with designed electrolytes of high Seebeck coefficient and ionic conductivity could result in record performance and is an interesting and important future perspective in the field of thermogalvanics. This work will advance the development of TGC toward practical applications and contribute to the field of low-grade heat harvesting.

## Supplementary Material



## Notes

Additional experimental details, materials, and methods, including photographs of experimental setup and complementary data (PDF)
