# Supplementary material for: Fully Printed Thermogalvanic Modules for Low-Grade Energy Harvesting
Source: ACS Appl Energy Mater. 2025 Aug 26;8(17):12868–77. doi: 10.1021/acsaem.5c02080 (PMC12421501; doi:10.1021/acsaem.5c02080)
Supplement: Supplementary file 1 [file ae5c02080_si_001.pdf]

Supporting Information

Fully Printed Thermogalvanic Modules for Low-Grade Energy Harvesting

Pedro Candiotto de Oliveira[1], Naveed ul Hassan Alvi[2], Najmeh Zahabi[1], Filippa Wentz[1,3], Kathrin Freitag[2], Lars Herlogsson[2], Ujwala Ail[1], Zia Ullah Khan[1], Igor Zozoulenko[1], Reverant Crispin[1], Dan Zhao[1*]*

[1] Laboratory of Organic Electronics, Department of Science and Technology, Linköping University, SE-601 74 Norrköping, Sweden.

[2] Printed, Bio and Organic Electronics Units, Department of Smart Hardware, Digital Systems Division, RISE Research Institutes of Sweden AB, Södra Grytsgatan 4, 602 33 Norrköping, Sweden.

[3] Wallenberg Wood Science Center, Department of Science and Technology, Linköping University, SE-601 74 Norrköping, Sweden

Corresponding authors: *reverant.crispin@liu.se, dan.zhao@liu.se.

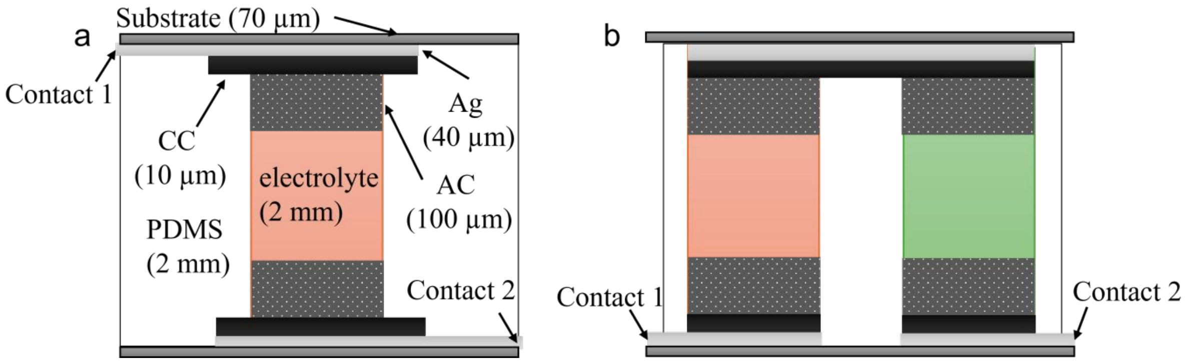

Figure S1. Illustration of the cross section of (a) single cell TGC and (b) p-n thermocouple of two cells.

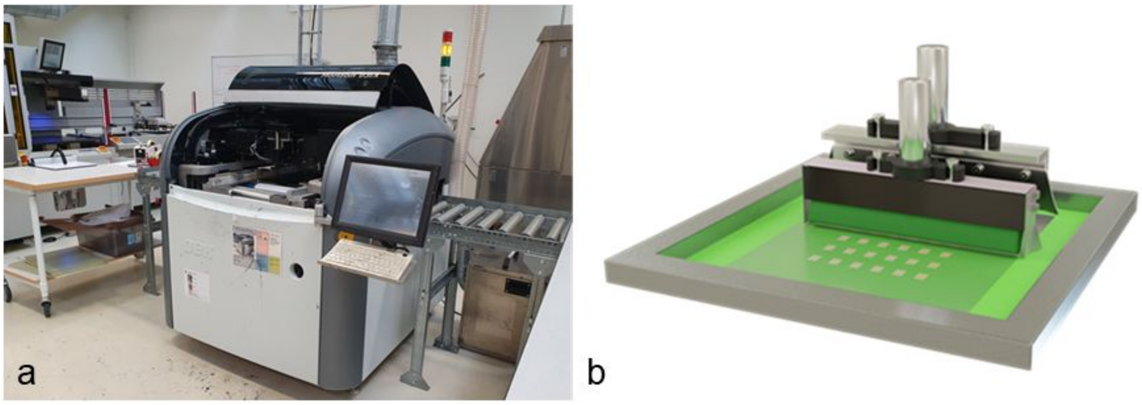

Figure S2. (a) A photo of screen printer DEK Horizon 03iX and (b) a schematic representation of the screen-printing process using a DEK screen printer.

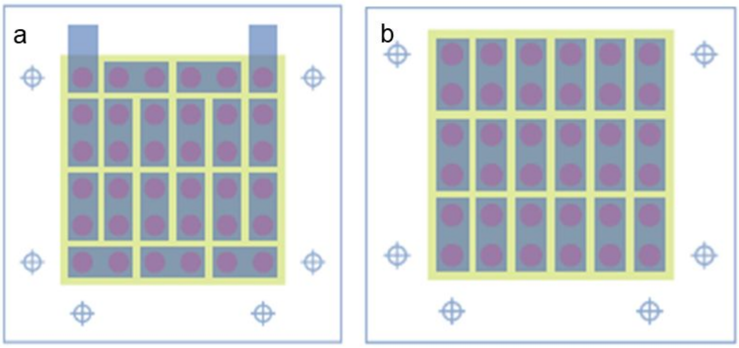

Figure S3. Scheme of the screen-printing masks of (a) bottom and (b) top side for printing TGM of 36 cells. The blue rectangles represent current collector, the purple circles represent electrodes and the yellow around represents UV-cured glue. All the layers were printed utilizing a DEK Horizon 03iX screen printer, which enables high-precision and repeatable printing. The resolution is also dependent on the screen-printing parameters, screen mesh count, ink rheology, printer settings and the materials used. For the TGM reported in this work, the printing resolution can reach 100 μm to 200 μm (Ag: 100μm, UV glue: 150μm, carbon composition and AC: 120μm). Layer alignment was maintained using the printer's built-in vision-based registration system, ensuring high accuracy between printed layers. We believe that the printing resolution does not limit the fill factor of the TGM. For the currently reported TGM, the limiting factor is the space between the chambers filled with electrolytes. When reducing the distance between the legs, electrolyte mixing was observed between the cells upon application of pressure. Hence, 4 mm between the cells covered by glue is used when filling liquid electrolytes, to guarantee good sealing and proper stability.

Table S1. Properties of materials involved in TGM material that are utilized in COMSOL simulations.

	**Thermal Conductivity ($W\ m^{-1}\ K^{-1}$)**	**Heat Capacity at Constant Pressure ($J\ kg^{-1}\ K^{-1}$)**	**Electrical Conductivity ($S\ m^{-1}$)**	**Density ($kg\ m^{-3}$)**	**Seebeck Coefficient ($V\ K^{-1}$)**	**Relative Permittivity**
**Copper**	400	384	59.6 E6	8920	1.5 E-6	Infinite
**PET**	0.1	2300	-	1175	-	3

**Silver**	430	233	6.3 E7	10490	1.0 E-6	Infinite
**Carbon Composition**	15	1000	92	2000	1.0 E-7	120
**Activated Carbon**	15	1000	56	2000	1.0 E-7	120
**UV-Cured Glue**	0.1	2300	-	640	-	3
**PMMA**	0.25	1400	5.8 E-8	1185	-	4.9
**$K_{3/4}[Fe(CN)_6]$**	0.611	3750	1.318	1000	-1.15 E-3	70
**$FeCl_{2/3}$**	0.611	3750	1.987	1000	1.08 E-3	70

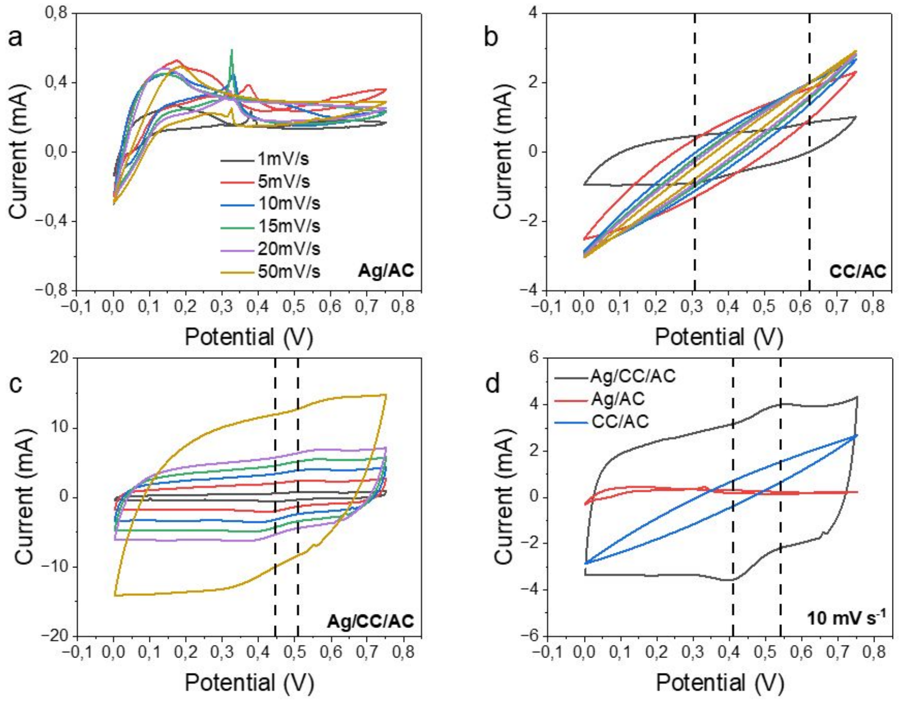

Figure S4. Electrochemical characterization of printed AC electrode on different current collectors in iron(II)/iron(III) chloride electrolyte. (a) Ag. (b) CC. (c) Ag/CC. (d) Comparison of the Ag/CC/AC, Ag/AC and CC/AC systems at 10 mV s^{-1} scan rate.

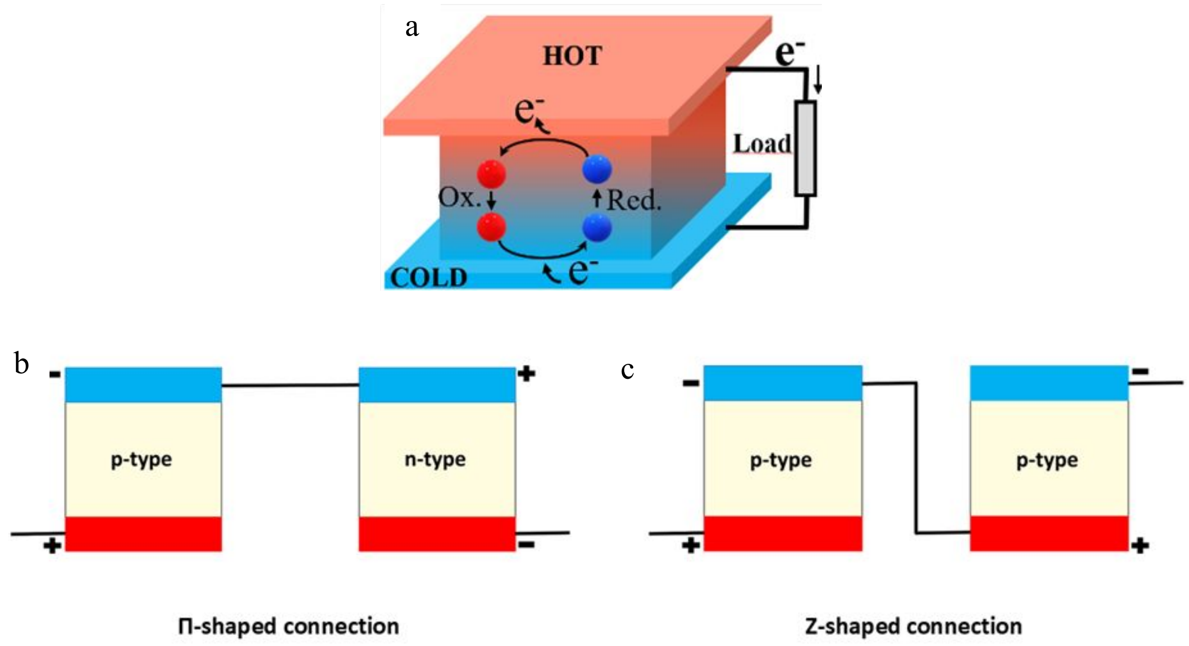

Figure S5. a) Illustration of the working principle of a thermogalvanic cell. By applying a temperature difference between the two electrodes, an output voltage is generated due to the entropy difference between the oxidized and the reduced species of a highly reversible redox couple (ΔS_{rc}). The Seebeck coefficient is the ratio between the generated thermovoltage and the temperature difference of the electrodes, defined by $Se = \Delta S_{rc}/nF$, where n is the number of electrons involved in the redox reaction and F is Faraday's constant. Constant power can be extracted from TGCs by connecting a load between the electrodes, while the temperature difference is being applied. b) and c) show the different connections between the TGCs in a TGM. The sign of the Seebeck coefficient is intrinsic to each redox couple, and their classification is between p-type (negative Seebeck coefficient) or n-type (positive Seebeck coefficient) electrolytes. For building thermogalvanic modules to obtain a useful practical voltage, individual cells must be connected electrically in series and thermally in parallel. The Π-shaped connection with alternating p and n-type electrolytes (Figure S5b) is simpler for

manufacturing then the Z-shaped connection , where only one type of electrolyte is utilized (Figure S5c).[1] For that reason, two standard p and n-type electrolytes were utilized in the present work.

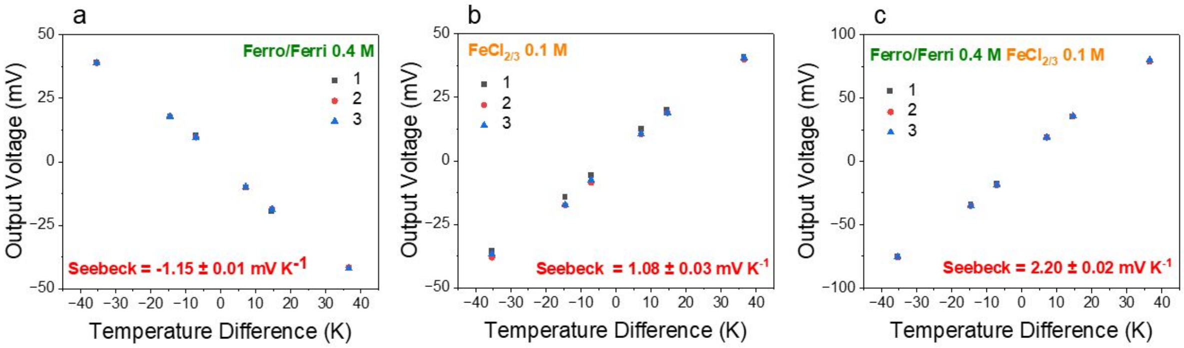

Figure S6. Applied temperature differences and obtained output voltages for (a) TGC of p-type electrolyte (ferro/ferricyanide) (b) TGC of n-type electrolyte (iron(II)/iron(III) chloride) (c) p-n thermocouple.

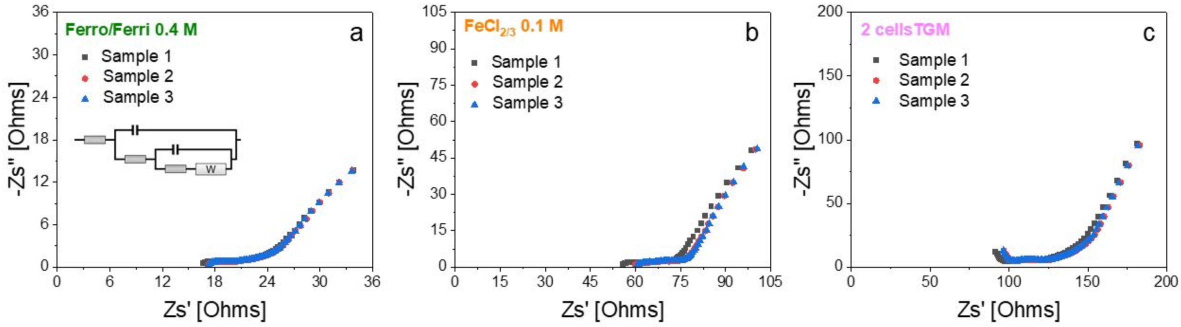

Figure S7. Nyquist plot of EIS spectra for (a) TGC of p-type electrolyte (b) TGC of n-type electrolyte (c) p-n thermocouple. The inset in (a) shows the equivalent circuit.

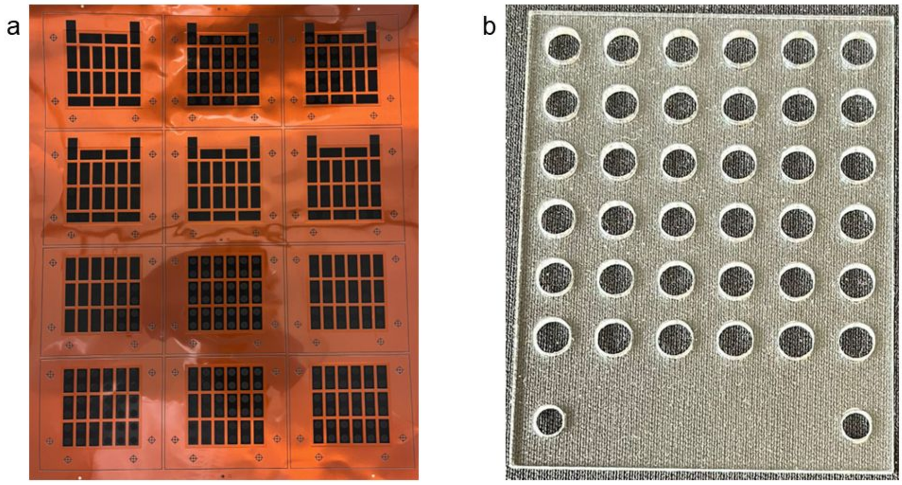

Figure S8. Photo of (a) screen-printed current collector and electrodes for the 36 cells TGM and (b) laser-drilled electrolyte chamber and separator PMMA with holes for alignment.

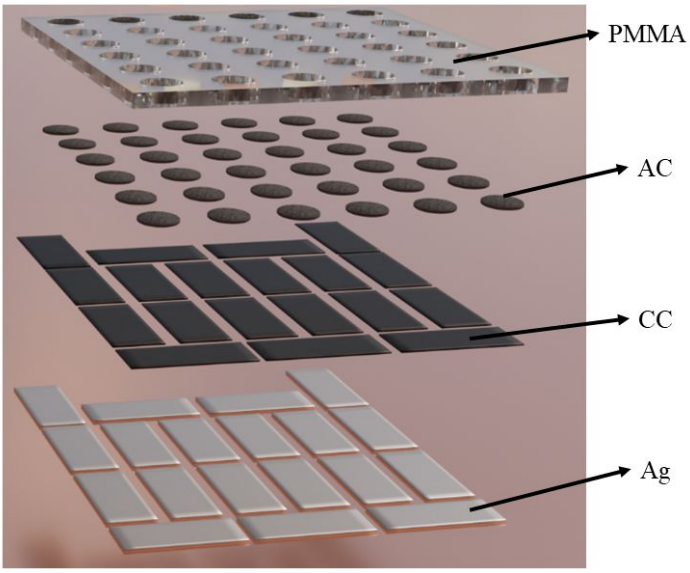

Figure S9. Cross section illustration of the printed layers of the TGM. The layers are printed over Skultuna copper substrate. The silver layer of the current collector is 10μm thick, the carbon composition is 6 μm thick, the AC electrode is 20 μm thick, the UV cured glue surrounding the electrodes with the same shape as the PMMA is 60 μm thick, and the PMMA spacer is 2 mm thick.

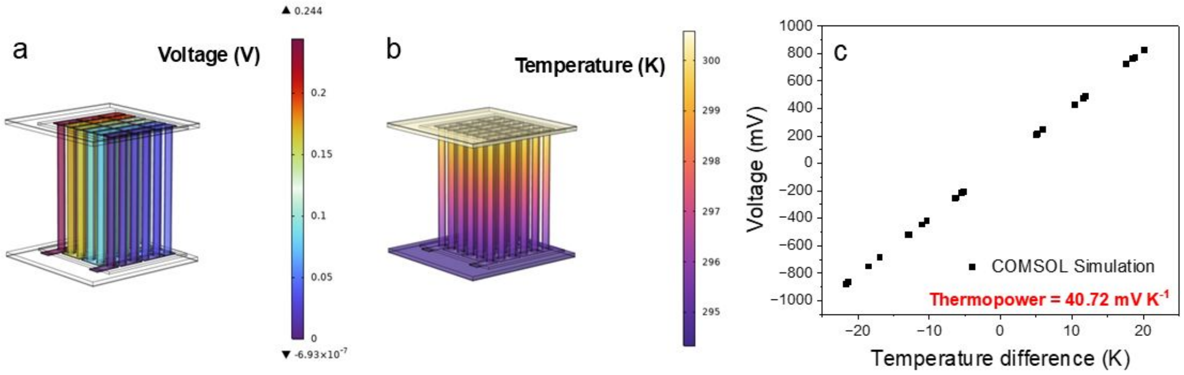

Figure S10. COMSOL simulated (a) voltage and (b) temperature difference across the TGM. (c) Simulated thermopower.

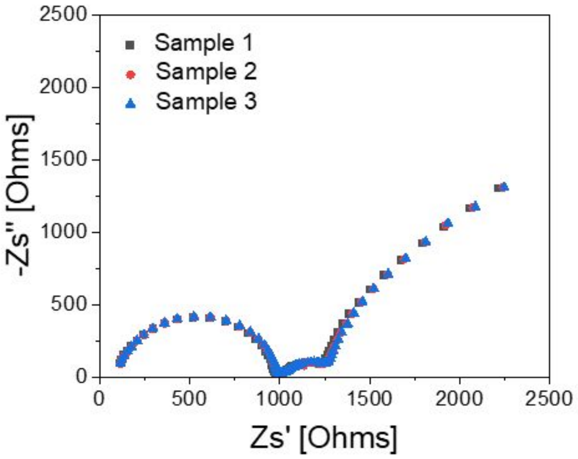

Figure S11. Nyquist plot of EIS spectra for 36 cells TGM. Based on these spectra, the contribution of different processes to the internal resistance of the TGM were estimated based on previous reports.[2,3] The two crosses of the first semicircle (at high frequency) on x-axis indicate the contact resistance and total series resistance (R_s), and the diameter of the first semicircle is related to the ionic resistance from the electrolyte. The second semicircle is related to the charge transfer resistance (R_{ct}), which can be estimated from the diameter. The last

semicircle at low frequency range represents the bulk resistance (R_{bulk}) that is related to the ionic transport in the bulk of electrode material.

Table S2. Different resistance components of TGCs with p and n-types of electrolytes, p-n thermocouple and TGMs. The values in parenthesis correspond to the standard deviation of the measurements. It is interesting to note that the series resistance (R_s) of the TGM is smaller than the sum of 18 p-n thermocouples (calculated). This is because only the ionic resistance scales up with the number of pairs, while the contact resistance does not increase thanks to the hybrid current collector with high conductivity. The charge transfer (R_{ct}) resistance obtained from the two methods show relatively similar magnitude. Meanwhile, the measured bulk resistance (R_{bulk}) of the module is much larger compared to the sum of 18 pairs of thermocouples. This could be due to the different limiting transportation in a single TGC and TGMs.

	**Ferro/Ferri (p-type)**	**$FeCl_{2/3}$ (n-type)**	**p-n thermocouple**	**Calculated 18 p-n**	**36 cells TGM**
**R_s (Ω)**	18.5 ± 0.3	63 ± 2	104 ± 3	1872	1010 ± 9
**R_{bulk} (Ω)**	2.43 ± 0.09	6.5 ± 0.1	16.8 ± 0.1	302.4	930 ± 33
**R_{ct} (Ω)**	2.56 ± 0.06	5.9 ± 0.2	14.0 ± 0.2	252	264 ± 3

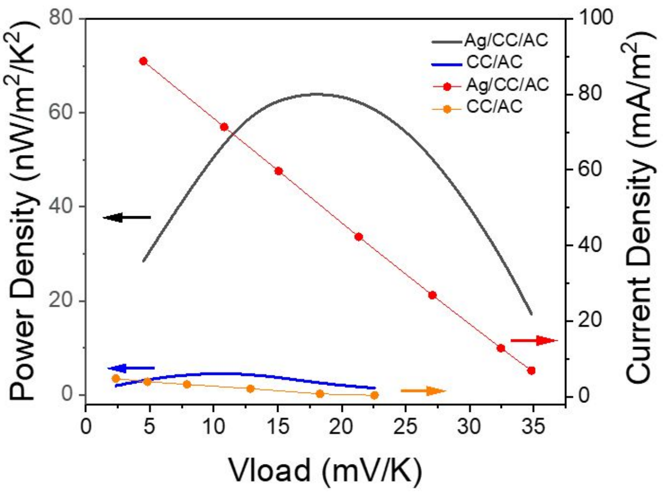

Figure S12. The comparison of TGMs with different current collectors.

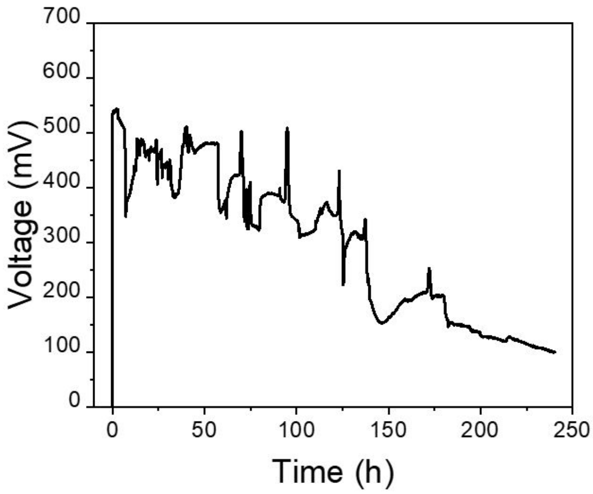

Figure S13. The stability of TGM with 36 cells for 240 hours with temperature gradient of 14 K.

Table S3. Comparison between TGMs reported in the literature and the one developed in this work. Regarding the electrolytes of the TGMs described in Table S3, the I^-/I_3^- and I^-/I_3/nanogels[4], $Cu(NO_3)_2$ + 91wt% sand grains,[5] $[Fe(CN)_6]^{3-/4-}$ + GdmCl[6] and FeCN-NH_4 and Fe-ClO_4[7] were modified and studied, bringing an increase in the thermopower reflected in the higher output

voltages. Only two of the studies reported power output (Duan et al., 2019[4], and Han et al. 2023[5]) of respectively 9μW and 240μW, for temperature differences of 10 K and 13 K, which corresponds, respectively, to 0.18 and 0.06 mW K^{-2} m^{-2} normalized power density, in the same range of our optimized printable TGM. Except for this work, all the others TGM are non-printable, which are difficult to enable automatic manufacturing and scaling-up.

**Electrode**	**Electrolyte**	**N° of cells**	**OCV (V)**	**SCC (μA)**	**Seebeck (mV K^{-1})**	**Power density (mW K^{-2} m^{-2})**	**Stability**	**Printable**	**REF**
Gold	I^-/I_3^- and I^-/I_3/nanogels	50	1.0	32	+0.71 -1.91	0.18	-	No	4
Copper	$Cu(NO_3)_2$ + 91wt% sand grains	36	2.0	600	+10.00	0.06	-	No	5
CNT/ PDMS	$[Fe(CN)_6]^{3-/4-}$ and Fe^{2+}/Fe^{3+}	12	0.0025	0,004	-1.05 +1.24		-	No	6
Graphite	$[Fe(CN)_6]^{3-/4-}$ + GdmCl	50	3.4	1200	+4.20		-	No	8
Graphite	FeCN-NH_4 and Fe-ClO_4	64	2.05	410	-1.29 +1.74		77% (7 days)	No	7
AC	$[Fe(CN)_6]^{3-/4-}$ and Fe^{2+}/Fe^{3+}	36	0.55	63	-1.15 +1.08	0.06	75% (4 days)	Yes	This work

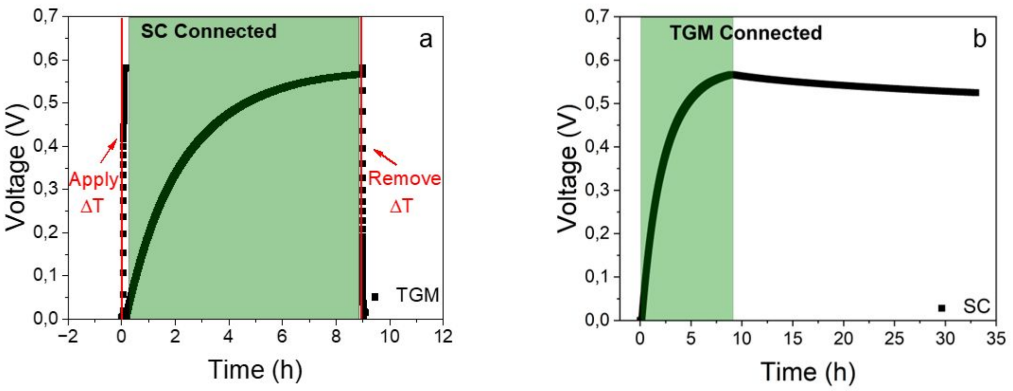

Figure S14. (a) The output voltage of a TGM while charging supercapacitor of 1.2 F (ΔT = 14 K). (b) The open circuit voltage of the same supercapacitor during charging and afterwards.

References:

(1) Liu, Y.; Cui, M.; Ling, W.; Cheng, L.; Lei, H.; Li, W.; Huang, Y. Thermo-Electrochemical Cells for Heat to Electricity Conversion: From Mechanisms, Materials, Strategies to Applications. *Energy Environ Sci* 2022, *15* (9), 3670–3687. https://doi.org/10.1039/d2ee01457b.

(2) Wijeratne, Kosala. Conducting Polymer Electrodes for Thermogalvanic Cells, 2018.

(3) Candiotto de Oliveira, P. S.; Ail, U.; Ullah Khan, Z.; Crispin, R.; Zhao, D. Printable Carbon-Based Thermogalvanic Cells. *Advanced Energy and Sustainability Research* 2025. https://doi.org/10.1002/aesr.202400428.

(4) Duan, J.; Yu, B.; Liu, K.; Li, J.; Yang, P.; Xie, W.; Xue, G.; Liu, R.; Wang, H.; Zhou, J. P-N Conversion in Thermogalvanic Cells Induced by Thermo-Sensitive

Nanogels for Body Heat Harvesting. *Nano Energy* 2019, *57*, 473–479. https://doi.org/10.1016/j.nanoen.2018.12.073.

(5) Han, K.; Xie, L. A Quasisolid Electrolyte Thermogalvanic Cell by Using Sand Grains. *Adv Electron Mater* 2023, *9* (7). https://doi.org/10.1002/aelm.202300089.

(6) Ding, T.; Zhou, Y.; Wang, X. Q.; Zhang, C.; Li, T.; Cheng, Y.; Lu, W.; He, J.; Ho, G. W. All-Soft and Stretchable Thermogalvanic Gel Fabric for Antideformity Body Heat Harvesting Wearable. *Adv Energy Mater* 2021, *11* (44). https://doi.org/10.1002/aenm.202102219.

(7) Kim, K.; Hwang, S.; Lee, H. Unravelling Ionic Speciation and Hydration Structure of Fe(III/II) Redox Couples for Thermoelectrochemical Cells. *Electrochim Acta* 2020, *335*. https://doi.org/10.1016/j.electacta.2020.135651.

(8) Duan, J.; Feng, G.; Yu, B.; Li, J.; Chen, M.; Yang, P.; Feng, J.; Liu, K.; Zhou, J. Aqueous Thermogalvanic Cells with a High Seebeck Coefficient for Low-Grade Heat Harvest. *Nat Commun* 2018, *9* (1). https://doi.org/10.1038/s41467-018-07625-9.
